# Supplementary material for: Heat adaptation of phage T7 under an extended genetic code
Source: Virus Evol. 2021 Dec 1;7(2):veab100. doi: 10.1093/ve/veab100 (PMC8923235; doi:10.1093/ve/veab100)
Supplement: veab100_Supp [file veab100_supp.zip › Sequencing_Analysis.zip!/Sequencing_Analysis/A1/evidence/MC_SIDE_2_49.html]

BRESEQ :: Evidence


| Missing coverage evidence... | | | | | | | | | | |
| --- | --- | --- | --- | --- | --- | --- | --- | --- | --- | --- |
|  |  |  | seq id | start | end | size | ←reads | reads→ | gene | description |
| \* | \* | ÷ | NC\_001604.1 | 1 | 108 | 108 | NA [0] | [1768] 1782 | *–/–* | –/– |

Only 100 of 1782 total aligned reads displayed.  

`‑‑‑‑‑‑‑‑‑‑‑‑‑‑TCTCACAGTGTACGGACCTAAAGTTCCCCCATAGGGGGTACCTAAAGCCCAGC‑CAATCACCTAAAGTCAACCTTCGGTTGACCTTGAGGGTTCCCTAAGGGTTGGGGATGACCCTTGGGTTTGTCTTTGGGTGTTACCTTGAGTGTCTCTCTGTGTCCCTATCTGTTACAGTCTCCTAAAGTATCCTCCTAAAGTCACCTCCTAACGTCCATCCTAAAGCCAACACCTAAAGCCTACACCTAAAGACCCATCAAGTCAACGCCTATCTTAAAGTTTAAACATAAAGACCAGACCTAAAGACCAGACCTAAAGACACTACATAAAGACCAGACCTAAAGACGCCTTGTTGTTAGCCATAAAGTGATAACCTTTAATCATTGTCTTTATTAATACAACTCACTATAAGGAG  >  NC_001604.1/1‑409`  

                                                                                                                           `|`                                                                                                                                                                                                                                                                                                              
`tcagagatgtgtaaaagagacaggtACGGACCTAAAGTTCCCCCATA‑GGGGTACCTAAAGACCAGC‑CAATCACCTAAAGTCAAACTTCGGTTGACCTTGAGGGTTCCCTAAGGGTTGGGGACGACCCTTGGGTTTGTCTTTGTGTGTTACCTTGAGTGTCTCTCTGTGTCCCTATATGTTACAGTCTCCTAAAGTATCCTCCTAAAGTCACCTCCTAACGTCCATCCTAAAGCCAACACCTAAAGCCTACACCTAAAGACCCATCAAGTAAACGCATATCTTAAAGTTTAAACAtaaagac                                                                                                                           <  1:171649/278‑1 (MQ=25)`  
`ccggagatgggtataagagacaggtACGGACCTAAAGTTCCCCCATA‑GGGGTACCTAAAGCCCAGC‑CAATCCCCTAAAGTCAACCTTCGGTTGACCTTGAGGGTTCCCTAAGGGTTGGGGATGACCCTTGGGTTTGTCTTTGGGTGTTACCTTGAGTGTCTCTCTGTGTCCCTATCTGTTACAGTCTCCTAAAGTATCCTCCTAAAGTCACCTCCTAACGTCCATCCTAAAGCCAACACCTAAAGCCTACACCTAAAGACCCATCAAGTCAACGCCTATCTTAAAGTTTAAACAtaaagac                                                                                                                           <  1:132724/278‑1 (MQ=25)`  
 `gggctcggagatgtgtataagagacaggACCTAAAGTTCCCCCATA‑GGGGTACCTAAAGCCCAGC‑CAATCACCTAAAGTCAACCTTCGGTTGACCTTGAGGGTTCCCTAAGGGTTGGGGATGACCCTTGGGTTTGTCTTTGGGTGTTACCTTGAGTGTCTCTCTGTGTCCCTATCTGTTACAGTCTCCTAAAGTATCCTCCTAAAGTCACCTCCTAACGTCCATCCTAAAGCCAACACCTAAAGCCTACACCTAAAGACCCATCAAGTCAACGCCTATCTTAAAGTTTAAACAtaaagacc                                                                                                                          <  1:385752/275‑1 (MQ=25)`  
      `cggatatgtgtataagagacaggACCTAAAGTTCCCCCATA‑GGGGTACCTAAAGCCCAGC‑AAATCACCTAAAGTCAACCTTCGGTTGACCTTGAGGGTTCCCTAAGGGTTGGGGATGACCCTTGGGTTTGTCTTTGGGTGTTACCTTGAGTGTCTCTCTGTGTCCCTATCTGTTACAGTCTCCTAAAGTATCCTCCTAAAGTCACCTCCTAACGTCCATCCTAAAGCCAACACCTAAAGCCTACACCTAAAGACCCATCAAGTCAACGCCTATCTTAAAGTTTAAACAtaaagaccagacc                                                                                                                     <  1:254122/280‑1 (MQ=255)`  
           `taagagacaggggtACGGACCTAAAGTTCCCCCATA‑GGGGTACCTAAAGTCCATC‑CAATCACCTAAAGTCAACCTTCGGTTGACCTTGAGGGTTCCCTAAGGGTTGGGGATGACCCTTGGGTTTGTCTTTGGGTGTTACCTTGAGTGTCTCTCTGTGTCCCTATCTGTTACAGTCTCCTAAAGTATCCTCCTAAAGTCACCTCCTAACGTCCATCCTAAAGCCAACACCTAAAGCCTACACCTAAAGACCCATCAAGTCAACGCCTATCTTAAAGTTTAAACAtaaagaccagacctaaag                                                                                                                <  1:190992/289‑1 (MQ=255)`  
           `taagagaacggtgtACGGACCTAAAGTTCCCCCATA‑GGGGTACCTAAAGCCCAGC‑CAATCACCTAAAGTGAACCTTCGGTTGACCTTGAGGGTTCCCTAAGTGTTGGGGATGACCCTTGGGTTTGTCTTTGGGTGTTACCTTGAGTGTCTCTCTGTGTCCCTATCTGTTACAGTCTCCTAAAGTATCCTCCTAAAGTCACCTCCTAACGTCCATCCTAAAGCCAACACCTAAAGCCTACACCTAAAGACCCATCAAGTCAACGCCTATCTTAAAGTTTAAACAtaaagaccagacctaaag                                                                                                                <  1:48120/291‑1 (MQ=255)`  
           `aaagagacagggggaCGGACCTAAAGTTCCCCCATA‑GGGGTACCTAAAGCCCAGC‑CAATTACCTAAAGTCAACCTTCGGTTGACCTTGAGGGTTCCCTAAGGGTTGGGGATGACCCTTGGGTTTGTCTTGGGGTGTTACCTTGAGTGTCTCTCTGTGTCCCTATCTGTTACAGTCTCCTAAAGTATCCTCCTAAAGTCACCTCCTAACGTCCATCCTAAAGCCAACACCTAAAGCCTACACCTAAAGACCCATCAAGTCAACGCCTATCTTAAAGTTTAAACAtaaagaccagacctaaag                                                                                                                <  1:469744/287‑1 (MQ=255)`  
              `tgtataagagtAAGGACCTAAAGTTCCCCCATA‑GGGGTACCTAAAGGCCCGC‑CAAGCACCTAAAGTCAACCTTCGGTTGACCTTGAGGGTTCCCTAAGGGTTGGGGATGACCCTTGGGTTTGTCTTTGGGTGTTACCTTGAGTGTCTCTCTGTGTCCCTATCTGTTACAGTCCCCTAAAGTATCCTCCTAAAGTCACCTCCTAACGTCCATCCTAAAGCCAACACCTAAAGCCTACACCTAAAGACCCATCAAGTCAACGCCTATCTTAAAGTTTAAACAtaaagaccagacctaaagacc                                                                                                             <  1:115401/292‑1 (MQ=255)`  
                   `caGTGTACGGACCTAAAGTTCCCCCATA‑GGGGTACCTAAAGCCCAGC‑CAATCACCTAAAGTCAACCTTCGGTTGACCTTGAGGGTTCACTAAGGGTTGGGGATGACCCTTGGGTTTGTCTTTGGGTGTTACCTTGAGTGTCTCTCTGTGTCCCTATCTGTTACAGTCTCCTAAAGTATCCTCCTAAAGTCACCTCCTAACGTCCATCCTAAAGCCAACACCTAAAGCCTAAACCTAAAGACCCATCAAGTCAAAGCCTATCTTAAAGTTTAAACAtaaagaccagacctaaagaccagacc                                                                                                        >  1:473357/1‑301 (MQ=255)`  
                   `aggtggACGGACCTAAATTGCCCCCATA‑GGGGTACCTAAAGCCCAGC‑CAATCACCTAAAGTCAACCTTCGGTTGACCTTGAGGGTTCCCTAAGGGGTGGGGATGACCCTTGGGTTTGTCTTTGGGTGTTACCTGGAGTGTCTCTCTGTGTCCCTATCTGTTACAGTCTCCTAAAGTATCCTCCTAAAGTCACCTCCTAACGTCCATCCTAAAGCCAACACCTAAAGCCTACACCTAAAGACCCATCAAGTCAACGCCTATCTTAAAGTTTAAACAtaaagaccagacctaaagaccagacc                                                                                                        <  1:106708/299‑1 (MQ=255)`  
                     `gtgtACTGACCTAAAGTTCCCCCATA‑GGGGTACCTAAAGCCCAGC‑CAATCACCTAAAGTCAACCTTCGGTTGACCTTGAGGGTTCCCTAAGGGTTGGGGATGACCCTTGGGTTTGTCTTTGGGTGTTCCCTTGAGTGTCTCTCTGTGTCCCTATCTGTTACAGTCTCCTAAAGTATCCTCCTAAAGTCACCTCCTAACGTCCATCCTAAAGCCAACACCTAAAGCCTACTACTTAAGACCCATCACGTCAACGCCTATCTTAACGTTTACACAtaaagaccaaacctaaagacctgtatct                                                                                                      >  1:110696/1‑294 (MQ=25)`  
                     `gtgtACGGACCTAAAGTTCCCCCATAGGGGGTACCTAAAGCCCAGC‑CAATCACCTAAAGTCAACCTTCGGTTGACCTTGAGGGTTCCCTAAGGGTTGGGGATGACCCTTGGGTTTGTCTTTGGGTGTTACCTTGAGTGTCTCTCTGTGTCCCTATCTGTTACAGTCTCCTAAAGTATCCTCCTAAAGTCACCTCCTAACGTCCATCCTAAAGCCACCACCTAAAGCCTAAACCTAAAGACCCATCAAGTCAACGCCTATCTTAAAGTTTAAACAtaaagaccagacctaaagacaagacca                                                                                                       >  1:261376/1‑300 (MQ=255)`  
                     `gtgtACGGACCTAAAGTTCCCCCATA‑GGGGTACCTAAAGCCCCGC‑CAATCACCTAAAGTAAACCTTCGGTTGACCTTGATGGTTCCCTAAGGGTTGGGGATTACCCTTGGGTTTGTCTTTGGGTTTTACCTTGAGTGTCTCTCTGTGTCCCTATCTGTTACAGTCTCCTAAAGTATCCTCCTACAGTCACCTCCTAACGTCCATCCTAAAGCCAACACCTAAAGCCTACACCTAACGACCCATCAAGTCAACGCCTACATTAACGTTTAAACAtacagacacgacctaaagaccctgtctc                                                                                                      >  1:87750/1‑294 (MQ=255)`  
                     `gtgtACGGACCTAAAGTTCCCCCATA‑GGGGTACCTAAAGCCCAGC‑CAATCACCTAAAGTCAACCTTCGGTTGACCTTGAGGGTTCCCTAATGGTTGGGGATGACCCTTGGGTTTGTCTTTGGGTGTTACCTTTAGTGTCTCTCTGTGTCCCTATCTGTTACAGTCTCCTAAAGTATCCTCCTAAAGTCACCTCCTAACGTCCATCCTATAGCCAACACCTAAAGCCTACAACTAAAGACCCATCAAGTCAACGCCTATCTTAAAGTTTAAACATAaagaccagaactaaagaccagaccta                                                                                                      >  1:209336/1‑301 (MQ=255)`  
                     `gtgtACGGACCTAAAGTTCCCCCATA‑GGGGTACCTAAAGCCCAGC‑CAATCACCTAAAGTCAACCTTCGGTTGACCTTGAGGGTTCCCTAAGGGTTGGGGATGACCCTTGGGTTTGTCTTTGGGTGTTACCTTGAGTGTCTCTCTGTGTCCCTATCTGTTACAGTCTCCTAAAGTCTCCTCCTAAAGTCACCTCCTAACGTCCATCCTAAAGCCAACACCTAAAGCCTACACCTAAAGACCCATCAAGTCAACGCCTATCTTAAAGTTTAAACATAaagaccagacctaaagaccagaccta                                                                                                      >  1:213337/1‑301 (MQ=255)`  
                     `gtgtACGGACCTAAAGTTCCCCCATA‑GGGGTACCTAAAGCCCAGC‑CAATCACCTAAAGTCAACCTTCGGTTGACCTTGAGGGTTCCCTAAGGGTTGGGGATGACCCTTGGGTTTGTCTTTGGGTGTTACCTTGAGTGTCTCTCTGTGTCCCTATCTGTTACAGTCTCCTAAAGTATCCTCCTAAAGTCACCTCCTAACGTCCATCCTAAAGCCAACACCTAAAGCCTACACCTAAAGACCCATCAAGTCAACGCCTATCTTACAGTTTACACATAaacaccagacctaaagcccagaccta                                                                                                      >  1:21741/1‑301 (MQ=255)`  
                     `gtgtACGGACCTAAAGTTCCCCCATA‑GGGGTACCTAAAGCCCAGC‑CAATCACCTAAAGTCAACCTTCGGTTGACCTTGAGGGTTCCCTAAGGGTTGGGGATGACCCTTGGGTTTGTCTTTGGGTGTTACCTTGAGTGTCTCTCTGTGTCCCTATCTGTTACAGTCTCCTAAAGTATCCTCCTAAAGTCACCTCCTAACGTCCATCCTAAAGCCAACACCTAAAGCCTACACCTAAAGACCCATCAAGTCAACGCCTATCTTAAAGTTTAAACATAaagaccagacctaaagaccagaccta                                                                                                      >  1:412943/1‑301 (MQ=255)`  
                     `gtgtACGGACCTAAAGTTCCCCCATA‑GGGGTACCTAAAGCCAAGC‑CAATCACCTAAAGTCAACCTTCGGTTGACCTTGAGGGTTCCCTAAGGGTTGGGGATGACCCTTGGGTTTGTCTTTGGGTGTTACCTTGAGTGTCTCTCTGTGTCCCTATCTGTTACAGTCTCCTAAAGTATCCTCCTAAAGTCACCTCCTAACGTCCATCCTAAAGCCAACACCTAAAGCATACACCTAAAGACCCATCAAGTCAACGCCTATCTTAAAGTTTAAACAtaaagaccagacctaaagaccggaccct                                                                                                      >  1:498/1‑299 (MQ=255)`  
                     `gtgtACGGACCTAAAGTTCCCACATAGGGGGTACCTAAAGCCCAGC‑CAATCACATAAAGTCAACCTTCGGTTGACCTTGAGGGTTCCCTAAGGGTTGGGGATGACCCTTGGGTTTGTCTTTGGGTGTTACCTTGAGTGTCTCTCTGTGTCCCTATCTGTTACAGTCTCCTAAAGTATCCTCCTAAAGTCACCTCCTAACGTCCATCCTAAAGCCCACACCTAAAGCCTACACCTAAAGACCCATCAAGTCAACGCCTATCTTAAAGTTTAAACATaaagaccagacctaaagaccagacct                                                                                                       >  1:477348/1‑301 (MQ=255)`  
                       `gtACGGACCTAAATTTCCCCCATA‑GGGGTACCTAAAGCCCAGC‑CAATCACCTAAAGTCAACCTTCGGTTGACCTTGAGGGTTCCCTAAGGGTT‑GGGATGACCCTTGGGTTTGTCTTTGGGTGTTACCTTGAGTGTCTCTCTGTGTTCCTATCTGTTACAGTCTCCTAAAGTATCCTCCTAAAGTAACCTCCTAACGTCCATCCTAAAGCCAACACCTAAAGCCTACACCTAAAGACCCATCAAGTCAACGCCTATCTTAAAGTTTAAACCTAAAgaccagacctaaagaccatacctcaaa                                                                                                   >  1:294139/1‑300 (MQ=255)`  
                       `gtACGGACCTAAAGTTTCCCCATA‑GGGGTACCTAAAGCCCAGC‑CAATCACCTAAAGTCAACCTTCGGTTGACCTTGAGGGTTCCCTAAGGGTTGGGGATGACCCTTGGGTTTGTCTTTGGGTGTTACCTTGAGTGTCTCTCTGTGTCCCTATCTGTTACAGTCTCCTAAAGTATCCTCCTAAAGTCACCTCCTAACGTCCATCCTAAAGCCAACACCTAAAGCCTACACCTAAAGACCCATCAAGTCAACGCCTATCTTAAAGTTTAAACATAAAgaccagacctaaagaccagacctaaa                                                                                                    >  1:51815/1‑301 (MQ=255)`  
                       `gtACGGACCTAAAGTTCCCCCATA‑GGGGTACCTAAAGCCCAGC‑CAATCACCTAAAGTCAACCTTCGGTTGACCTTGAGGGTTCCCTAAGGGTTGGGGATGACCCTTGGGTTTGTCTTTGGGTGTTACCTTGAGTGTCTCTCTGTGTCCCTATCTGTTACAGTCTCCTAAAGTATCCTCATAAAGTCACCTCCTAACGTCCATCCTAAAGCCAACACCTAAAGCCTACACCTAAAGACCCATCAAGTCAACGCCTATCTTAAAGTTTAAACATAAAgaccagacctaaagaccagacctaaa                                                                                                    >  1:104953/1‑301 (MQ=255)`  
                       `gtACGGACCTAAAGTTCCCCCATA‑GGGGTACCTAAAGCCCAGC‑CAATCACCTAAAGTCAACCTTCGGTTGACCTTGAGGGTTCCCTAA‑GGTTGGGGATGACCCTTGGGTTTGTCTTTGTGTGTTACCTTGCGTGTCTCTCTGTGTCCCTATCTGTTACAGTCTCCTAAAGTATTCTCCTACAGTCACCTCCTACCGTCCATCCTAAAGCCAACTCCTAAAGCCTACACCTAAAGACCCATCCATTCACCGCCTATCTTAACGTTTAATCATAAAgaccagacctacagaccagccctaaaa                                                                                                   >  1:230251/1‑300 (MQ=25)`  
                        `tACGGACCTAAAGTTCCCCCAGA‑GGGGTACCTAAAGGCCAGC‑CAATCACCTAAAGTCAACCTTCGGTTGACCTTGAGGGTTCCCTAAGGGTTGGGGATGACCCTTGGGTTTGTCTTTGGGTGTTACCTTGAGTGTCTCTCTGTGTCCCTATCTGTTACAGTCTCCTAAAGTATCCTCCTAAAGTCACCTCCTAACGTCCATCCTAAAGCCAACACCTAAAGCCTACACCTAAAGACCCATCAAGTCAACGCCTATCTTAAAGTTTAAACATAAAGaccagacctaaagaccagacctaaag                                                                                                   <  1:63113/301‑1 (MQ=255)`  
                          `cGGACCTAAAGTTCCCACATA‑GGGGTCCCTAAAGCCCAGC‑CAATCACCTAAATTCAACCTTCGGTTGTCCTTGAGGGTTCCCTAAGGGTTGGGGATGACCCTTGGGTTTGTCTTTGGGTGTTACCTTGAGTGTCTCTCTGTTTCCCTATCTGTTACAGTCTCCCAAAGTATCCTCCTAAAGTCACCTCCTAACGTCCATCCTAAAGCCAACACCTAAAGCCTACACCTAAAGACCCATCAAGTCAACGCCTATCTTAAAGTTTAAACATAAAGACcagacctaaagaccagacctaaagac                                                                                                 <  1:233607/301‑1 (MQ=255)`  
                          `aggATCTAAAGTTCCCCCATA‑GGGGTACCTAAAGCCCAGC‑CAATCACCTAAAGTCAACCTTCGGTTGACCTTGAGGGTTCCCTAAGGGTTGGGGATGACTCTTGGGTTTGTCTTTGGGTGTTACCTTGAGTGTATCTCTGTGTCCCTATCTGTTACAGTCTCCTAAAGTATCCTCCTAAAGTAACCTCCTAACGTCCATCCTAAAGCCAACACCTAAAGCCTACACCTAAAGACCCATCAAGTCAACGCCTATCTTAAAGTTTAAACATAAAGACcagacctaaagaccagacctaaagac                                                                                                 <  1:300143/300‑1 (MQ=255)`  
                            `gACCTAAAGTTCCCCCATA‑GGGGTACCTAAAGCCCAGC‑CAATCACCTAAAGTCAACCTTCGGTTGACCTTGAGGGTTCCCTAAGGGTTGGGGATGACCCTTGGGTTTGTCTTTGGGTGTTACCTTGAGTGTCTCTCTGTGTCCCTATCTGTTACAGTCTCCTAAAGTATCCTCCTAAAGTCACCTCCTAACGTCCATCCTAAAGCCAACACCTAACGCCTACACCTAAAGACCCATCAAGTCAACGCCTATCTTCAAGTTTCAACAtaacgaccagccctaacaaccctgtctcttataca                                                                                               >  1:369330/1‑282 (MQ=25)`  
                            `gACCTAAAGTTCCCCCATA‑GGGGTACCTAAAGCCCAGC‑CAATCACCTAAAGTCAACCTTCGGTTGACCTTGAGGGTTCCCTAAGGGTTGGGGATGACCCTTGGGTTTGTCTTTGGGTGTTACCTTGAGTGTCTCTCTGTGTCCCTATCTGTTACAGTCTCCTAAAGTATCCTCCTAAAGTCACCTCCTAACGTCCATCCTAAAGCCAACACCTAAAGCCTACACCTAAAGACCAATCAAGTCAACGACTATCTTAAAGTTTAAACATAAAACCcagacctaaagaccagacctaaagactt                                                                                               >  1:144811/1‑299 (MQ=255)`  
                            `gACCTAAAGTTCCCCCATA‑GGGGTACCTAAAGCCCAGC‑CAATCACCTAAAGTCAACCTTCGGTTGACCTTGAGGGTTCCCTAAGGGTTGGGGATGACCCTTGGGTTTGTCTTTGGGTGTTACCTTGAGTGTCTCTCTGTGTCCCTATCTGTTACAGTCTACTAAAGTATCCTCCTAAATTCACCTCCTAACGTCCATCCTCAAGCCAACACCTAAAGCCTACACCTAAAGACCCATCACGTCAACGCCTATCTTAAAGTTTAAACATAAAGACCCGACCTAAAGACCAGACCTACAGacac                                                                                               >  1:349884/1‑301 (MQ=255)`  
                            `ataagagacagcccccATA‑GGGGTACCTAAAGCCCAGC‑CAATCACCTAAAGTCAACCTTCGGTTGACCTTGAGGGTTCCCTAAGGGTTGGGGATGACCCTTGGGTTTGTCTTTGGGTGTTACCTTGAGTGTCTCTCTGTGTCCCTATCTGTTACAGTCTCCTAAAGTATCCTCCTAAAGTCACCTCCTAACGTCCATCCTAAAGCCAACACCTAAAGCCTACACCTAAAGACCCATCAAGTCAACGCCTATCTTAAAGTTTAAACATAAAGACCAGACCTAAAGACCAGACCTAAAGacac                                                                                               <  1:93813/290‑1 (MQ=255)`  
                             `aCCTAAAGTTCCCCCATA‑AGGGTACCTAAAGCCCAGC‑CAATCACCTAAAGTCAACCTTCGGTTGACCTTGAGGGTTCCCTAAGGGTTGGGGATGACCCTTGGGTTTGTCTTTGGGTGTTACCTTGAGTGTCTCTCTGTGTCCCTATCTGTTACAGTCTCCTAAAGTATCCTCCTAAAGTCACCTCCTAACGTCCATCCTAAAGCCAACACCTAAAGCCTACACCTAAAGACCCATCAAGTCAACGCCTATCTTAAAGTTTAAACATAAAGACCAGACCTAAAGACCAGACCTAAAGacaat                                                                                              >  1:241988/1‑299 (MQ=255)`  
                              `ccTAAAGTTCCCCCATA‑GGGGTACCTAAAGCCCAGC‑CAATCACCTAAAGTCAACCTTCGGTTGACCTTGAGGGTTCCCTAAGGGTTGGGGATGACCCTTGGGTTTGTCTTTGGGTGTTACCTTGAGTGTCTCTCTGTGTCCCTATCTGTTACAGTCTCCTAAAGTATCCTCCTAAAGTCACCTCCTAACGTCCATCCTAAAGCCAACACCTAAAGCCTACACCTAAAGACCCATCAAGTCAACGCCTATCTTAAAGTTTAAACATAAAGACCAGACCTAAAGACCAGACCTAAAGGCACTa                                                                                             >  1:164886/1‑301 (MQ=255)`  
                               `cTAAAGTTCCCCCATA‑GGGGTACCTAAAGCCCAGC‑CAATCCCCTAGAGTCAACCTTCGGTTGACCTTGAGGGTTCCCTAAGGGTTGGGGATTACCCTTGGGTTTGTCTTTGGGTGTTACCTTGAGTGTCTCTCTGTTTCCCTATCTGTTACAGTCTCCTAAAGTATCCTCCTAAAGTCACCTCCTAACGTCCATCCTAAAGCCAACACCTAAAGCCTACACCTAAAGACCCATCAAGTCAACGCCTATCTTAAAGTTTAAACATAAAGACCAGACCTAAAGACCAGACCTAAAGACACTAc                                                                                            <  1:342074/301‑1 (MQ=255)`  
                                `tAAAGTTCCCCCATA‑GGGGTACCTAAAGCCCAGC‑CAATCACCTAAAGTCAACCTTCGGTTGACCTTGAGGGTTCCCTAAGGGTTGGGGATGACCCTTGGGTTTGTCTTTGGGTGTTACCTTGAGTGTCTCTCTGTGTCCCTATCTGTTACAGTCTCCTAAAGTATCCTCCTAAAGTCACCTCCTAACGTCCATCCTAAAGCCAACACCTAAAGCCTACACCTAAAGACCCATCAAGTAAACGCCTATCTTAAAGTTTAAACATAAAGACCAGACCTAAAGACCAGACCTAAAGACACTACa                                                                                           <  1:427391/301‑1 (MQ=255)`  
                                  `atatgtgtataatatacagaCCTAAAGCCCAGC‑CAATCACCTAAAGTCAACCTTCGGTTGACCTTGAGGGTTCCCTAAGGGTTGGGGATGACCCTTGGGTTTGTCTTTGGGTGTTACCTTGAGTGTCTATCTGTGTCCCTATCTGTTACAGTATCCTAAAGTATCCTCCTAAAGTCACCTCCTAACGTCCATCCTAAAGCCAACACCTAAAGCCTACACCTAAAGACCCATCAAGTCAACGCCTATCTTAAAGTTTAAACATAAAGACCAGACCTAAAGACCAGACCTAAAGACACTACAt                                                                                          <  1:176025/282‑1 (MQ=255)`  
                                      `ccccccATA‑GGGGTACCTAAAGCCCAGC‑CAATCGCCTAAAGTCAACCTTCGGTTGACCTTGAGGGTTCCCTAAGGGTTGTGGATGACCCTTGGGTTTGTCTTTGGGTGTTACCTTGAGTGTCTCTCTGTGTCCCTATCTGTTACAGTCTCCTAAAGTATCCTCCTAAAGTCACCTCCTAACGTCCATCCTAAAGCCAACACCTAAAGCCTACACCTAAAGACCCATCAAGTCAACGCCTATCTTAAAGTTTAAACATAAAGACCAGACCTAAAGACCAGACCTAAAGACACTACATAAaga                                                                                     <  1:33000/300‑1 (MQ=255)`  
                                       `cccccATA‑GGGGTACCTAAAGCCCAGC‑CAATCACCTAAAGTCAACCTTCGGTTGACCTTGATGGTTCCCTAAGGGTTGGGGATGACCCTTGGGTTTGTCTTTGGGTGTTACCTTGAGTGTCTCTCTGTGTCCCTATCTGTTACAGTCTCCTAAAGTATCCTCCTAAAGTCACCTCCTAACGTTCATCCTAAAGCCAACACCTAAAGCCTACACCTAAAGACCCATCAAGTCAACGCCTATCTTAAAGTTTAAACATAAAGaccagccctaaagaccagacctaaagcaccctgtctcttat                                                                                    >  1:58483/1‑286 (MQ=255)`  
                                       `cccccATA‑GGGGTACCTAAAGCCCAGC‑CAATCACCTAAAGTCAACCTTCGGTTGACCTTGAGGGTTCCCTAAGGGTTGGGGATGACCCTTGGGTTTGTCTTTGGGTGTTCCCTTGAGTGTCTCTCTGTGTCCCTATCTGTTACAGTCTCCTAAAGTATCCTCCTAAAGTCACCTCCTAACGTCCATCCTAAAGCCAACACCTAAAGCCTACACCTAAAGACCCATCAAGTCAACGCCTATCTTAAAGTTTAAACATAAAGACCAGACCTAAAGACCAAACCTAAAGACACtcactcaagct                                                                                    >  1:325180/1‑291 (MQ=255)`  
                                       `cccccATA‑GGGGTACCTAAAGCCCAGC‑CAATCACCTAAAGTCAACCTTCGGTTGACCTTGAGGGTTCCCTAAGGGTTGGGGATGACCCTTGGGTTTGTCTTTGGGTGTTACCTTGAGTGTCTCTCTGTGTCCATATCTGTTACAGTCTCCTAAAGTATCCTCCTAAAGTCACCTCCTAACGTCCATCCTAAAGCCAACACCTAAAGCCTACACCTAAAGACCCATCAAGTCAACGCCTATCTTAAAGTTTAAACATAAAGACCAGACCTAAAGACCAGACCTAAAGACACTACATAAagac                                                                                    >  1:446611/1‑301 (MQ=255)`  
                                       `cccccATA‑GGAGTACCTACAGCCCAGC‑CAATCACCTAAAGTCAACCTTCTGTTGACCTTGAGGGTTTCCTAAGGGTTTGTGATGACCCTTGGGTTTGTCTTTGGGTTTTACCTTGAGTGTCTCTCTGTGTCCCTATCTGTTACAGTCTCCTATAGTATCCTCCTAAAGTCACCTCCTAACGTCCATCCTAAAGCCAACACCTAAAGCCTACACCTAAAGACCCATCAAGTCAATGCCTATCTTAAAGTTTAAACATAAAGACCAGACCTAAAGACCAGACCTAAAGACACTACATAAagac                                                                                    <  1:354022/301‑1 (MQ=255)`  
                                       `agcccATA‑GGGGTACCTAAAGCCCAGC‑CAATCACCTAAAGTCAACCTTCGGTTGACCTTGAGGGTTCCCTAAGGGTTGGGGATGACCCTTGGGTTTGTCTTTGGGTGTTACCTTGAGTGTCTCTCTGTGTCCCTATCTGTTACAGTCTCCTAAAGTATCCTCCTAAAGTCACCTCCTAACGTCCATCCTAAAGCCAACACCTAAAGCCTACACCTAAAGACCCATCAAGTCAACGCCTATCTTAAAGTTTAAACATAAAGACCAGACCTAAAGACCAGACCTAAAGACACTACATAAagac                                                                                    <  1:420730/299‑1 (MQ=255)`  
                                        `tgtataagagacagccTAAAGCCCAGC‑CAATCACCTAAAGTCAACCTTCGGTTGACCTTGAGGGTTCCCTAAGGGTTGGGGATGACCCTTGGGTTTGTCTTTGGGTGTTACCTTGAGAGTCTCTCTGTGTCCCTAACTGTTACAGTCTACTAAAGTATCCTCCTAAAGTCACCTCCTAACGTCCATCCTAAAGCCAACACCTAAAGCCTACACCTAAAGACCCATCAAGTCAACGCCTATCTTAAAGTTTAAACATAAAGACCAGACCTAAAGACCAGACCTAAAGACACTACATAAagac                                                                                    <  1:118730/287‑1 (MQ=255)`  
                                        `ccccgatagtggTACCTAAAGCCCAGCACAAT‑ACCTATAGTAAACCTTCGGTTGACCTTGAGGGTTCCCTAAGGGTTGGGGATGACCCTTGGGTTTGTCTTTGGGTTTTACCTTTAGTGTCTCTCTGTTTCCCTATCTGTTACAGTCTCCTAAAGTATCCTCCTAAAGTCACCTCCTAACGTCCATCCTAAAGCCAACCCCTAAAGCCTACACCTAAAGACCCATCAAGTCAACGCCTATCTTAAAGTTTAAACATAAAGACCAGACCTAAAGACCAGACCTAAAGACACTACATAAagac                                                                                    <  1:67821/291‑1 (MQ=37)`  
                                         `cccATA‑GGGGTACCTAAAGCCCAGC‑CAATCACCTAAAGTCAACCTTCGGTTGACCTTGAGGGTTCCCTAAGGGTTGGGGATGACCCTTGGGTTTGTCTTTGGGTGTTACCTTGAGTGTCTCTCTGTGACCCTATCTGTTACAGTCTCCTAAAGTATCCCCCTAAAGTCAACTCCTAACGTCCATTCTAAAGCCAACACCTAAAGCCTACACCTAAAGACCTATCCATTCAACGCCTATTTTAAAGTTTAAACATAAGGACCATACCTAAAGACCAGACCTAAAGacacctgtctcttataa                                                                                  >  1:337990/1‑288 (MQ=25)`  
                                          `ccATA‑GGGGTACCTAAAGCCCAGC‑CAATCACCTAAAGTCAACCTTCGGTTGACCTTGAGGGTTCCCTAAGGGTTGGGGATGACCCTTGGGTTTGTCTTTGGGTGTTACCTTGAGTGTCTCTCTGTGTCCCTATCTGTTACAGTCTCCTAAAGTGTCCTCCTAAAGTCACCTCCTAACGTCCATCCTAAAGCCAACACCTAAAGCCTACACCTAAAGAACCATCAAGTCAACGCCTATCTTAAAGGTTAAACATAAAGACCAGACCTAACGACCAGACCTAAAGACACTACATAAagaccaa                                                                                 >  1:224663/1‑300 (MQ=255)`  
                                           `ccatatgggTACCTAAAGCCCAGC‑CAATCACCTAAAGTCAACCTTCGGTTGACCTTGAGGGTTCCCTAAGGGTTGGGGATGACCCTTGGGTTTGTCTTTGGGTGTTACCTTGAGTGTCTCTCTGTGTCCATATCTGTTACAGTCTCCTAAAGTATCCTCCTAAAGTCACCTCCTAACGTCCATCATAAAGCCAACACCTAAAGCCTACACCTAAAGACCCATCAAGTCAACGCCTATCTTAAAGTTTAAACATAAAGACCAGACCTAAAGACCAGACCTAAAGACACTACATCAagaccaa                                                                                 >  1:221478/7‑300 (MQ=255)`  
                                              `taggggTACCTAAAGCCCAGC‑CAATCACCTAAAGTCAACCTTCGGTTGACCTTGAGGGTTCCCTAAGGGTTGGGGATGACCCTTTGGTTTGTCTTTGGGTGTTACCTTGAGTGTCTCTCTGTGTCCCTATCTGTTACAGTCTCCTAAAGTATCCTCCTAAAGTCACCTCCTAACGTCCATCCTAAAGCCAACACCTAAAGCCTACACCTAAAGACCCATCAAGTCAACGCCTATCTTAAAGTTTAAACATAAAGACCAGACCTAAAGACCAGACCTAAAGACACTACATAAagaccagacc                                                                              <  1:404492/299‑1 (MQ=255)`  
                                              `taggggTACCTAAAGCCCAGC‑CAATCACCTAAAGTCAACCTTCGGTTGACCTTGAGGGTTCCCTAAGGGTTGGGGATGACCCTTGGGTTTGTATTTGGGTGTTACCTTGAGTGTCTCTCTGTGTCCCTATCTGTTACAGTCTCCTAAAGTATCCTCCTAAAGTCACCTCCTAACGTCCATCCTAAAGCCAACACCTAAAGCCTACACCTAAAGACCCATCAAGTCAACGCCTATCTTAAAGTTTAAACATAAAGACCAGACCTAAAGACCAGACCTAAAGACACTACATAAagaccagacc                                                                              >  1:441809/3‑301 (MQ=255)`  
                                                `ggggTACCTAAAGCCCAGC‑CAATCACCTAAAGTCAACCTTCGGTTGACCTTGAGGGTTCCCTAAGGGTTGGGGATGACCCTTGGGTTTGTCTTTGGGTTTTACCTTGATTGTCTCTCTGTGTCCCTATCTGTTACAGTCTCCTAAAGTATCCTCCTAAAGTCACCTCCTAACGTCCATCCTAAAGCCAACACCTAAAGCCTACACCTAAAGCCCCATCAAGTCAACGCCTATCTTAAAGTTTAAACATAAAGACCAGACCTACAGACCACACCTAAAGACACTACATAAAGACCATACCTa                                                                            >  1:78197/1‑301 (MQ=255)`  
                                                 `catatggctagcatgactggtggacagcaaaGTCAACCTTCGGTTGACCTTGAGGGTTCCCTAAGGGTTGGGGATGACCCTTGGGTTTGTCTTTGGGTGTTACCTTGAGTGTCTCTCTGTGTCCCTATCTGTTACAGTCTCCTAAAGTATCCTCCTAAAGTCACCTCCTCACGTCCATCCTAAAGCCAACACCTAAAGCCTACACCTAACGACCCATCAAGTCAACGCCTATCTTAAAGTTTAAACATAAAGACCAGACCTAAAGACCAGACCTAAAGACACTACATAAAGACCAGACCTa                                                                            >  1:365343/29‑301 (MQ=38)`  
                                                    `tACCTAAAGCCCAGC‑CAATCACCTAAAGTCAACCTTCGGTTGACCTTGAGGGTTCCCTAAGGGTTGGGGATGACCCTTGGGTTTGTCTTTGGGTGTTACCTTGAGTGTCTCTCTGTGTCCCTATCTGTTACAGTCTCCTAAAGTATCCTCCTAAAGTCACCTCCTAACGTCCATCCTAAAGCCAACACCTAAAGCCTACACCTAAAGACCCATCAAGTCAACGCCTATCTTAAAGTTTAAACATAAAGACCAGACCTAAAGACCAGACCTAAAGACACTACATAAAGACCAGACCTAAAGa                                                                        <  1:100356/301‑1 (MQ=255)`  
                                                     `aCCTAAAGCCCAGC‑CAATCACCTAAAGTCAACCTTCGGTTGACCTTGAGGGTTCCCTAAGGGTTGGGGATGACCCTTGGGTTTGTCTTTGGGTGTTACCTTGAGTGTCTCTCTGTGTCCCTATCTGTTACAGTCTCCTAAAGTATCCTCCTAAAGTCACCTCCTAACGTCCATCCTAAAGCCAACACCTAAAGCCTACACCTAAAGACCCATCAAGTCAACGCCTATCTTAAAGTTTAAACATAAAGACCAGACCTAAAGACCAGAACTAAAGACACTACATAAAGACCAGACCTAAAGAc                                                                       >  1:153200/1‑301 (MQ=255)`  
                                                     `aCCTAAAGCCCAGC‑CAATCACCTAAAGTCAACCTTCGGCTGACCTTGAGGGTTCCCTAAGGGTTGGGGATGACCCTTGGGTTTGTCTTTGGGTGTTACCTTGAGTGTCTCTCTGTGTCCCGATCTGTTACAGTCTCCTAAAGTACCCTCCTAAAGTCACCTCCTAACGTCCATCCTAAAGCCAACACCTAAAGCATACACCTAACGACCCATCAAGTCAACGCCTATCTTAAAGTTTAAACATAAAGACCAGACCTAAAGACCAGACCTAAAGACACTAAATAAAGACCAAACATAAAGaa                                                                       >  1:185219/1‑300 (MQ=255)`  
                                                      `ccTAAAGCCCAGC‑CAATCACCTAAAGTCAACCTTCGGTTGACCTTGAGGGTTCCCTAAGGGTTGGGTATGACCCTTGGGTTTGTCTTTGGGTGTTACCTTGAGTGTCTCTCTGTGTCCCTATCTGTTACAGTCTCCTAAAGTATCCTCCTAAAGTCACCTCCTAACGTCCATCCTAAAGCCAACACCTAAAGCCTACACCTAAAGACCCATCAAGTCACCGCCTATCTTAAAGTTTAAACATAACGACCAGACCTAAAGACCAGACCTACAGACACTACATAAagacctgtctcttataaa                                                                      >  1:360080/1‑288 (MQ=255)`  
                                                      `ccTAAAGCCCAGC‑CAATCACCTAAAGTCAACCTTCGGTTGACCTTGAGGGTTCCCTAAGGGTTGGGGATGACCCTTGGGTTTGTCTTTGGGTGTTACCTTGAGTGTCTCTCTGTGTCCCTATCTGTTACAGTCTCCTAAAGTATCCTCCTAAAGTCACCTACTAACGTCCATCCTAAAGCCAACACCTAACGCCTACAACTAAAGACCCATCAAGTCAACGCCTATCTTAAAGTTTAAACATAAAGACCAGACCTAAAGACCAGACCTAAAGACCCTACATAAagaccagaaccactccct                                                                      >  1:437701/1‑291 (MQ=255)`  
                                                            `gCCCAGC‑CAATCACCTAAAGTCAACCTTCGGTTGACCTTGAGGGTTCCCTAAGGGTTGGGGATGACCCTTGGGTTTGTCTTTGGGTGTTACCTTGAGTGTCTCTCTGTGTCCCTATCTGTTACAGTCTCCTAAAGTATCCTCCTAAAGTCACCTCCTAATGTCCATCCTAAAGCCAACACCTAAAGCCTACACCTAAAGACCCATCAAGTCAACGCCTATCTTAAAGTTTAAACATAAAGACCAGACATAAAGACCAGACATAAAGACACTACATAAAGACCAGAACTAAAgctgtccctt                                                                >  1:451065/1‑292 (MQ=255)`  
                                                            `accaaGC‑CAAGCACCTAAAGTCAACCTTCGGTTGACCTTGAGGGTTCCCTAAGGGTTGGTGATGACCCTTGGGTTTGTCTTTGGGTGTTACCTTGAGTGTCTCTCTGTGTCCCTATCTGTTACAGTCTCCTAAAGTATCCTCCTACAGTCACCTCCTAACGTCCATCCTAAAGCCAACACCTAAAGCCTACACCTAAAGACCCATCAAGTCAACGCCTATCTTAAAGTTTAAACATAAAGACCAGACCTAAAGACCAGACCTAAAGACACTACATAAAGACCAGACCTAAAGACGCCttgt                                                                <  1:463726/297‑1 (MQ=255)`  
                                                                 `gC‑CAATCACCTAAAGTCAACCTTCGGTTGACCTTGAGGGTTCCCTAAGGGTTGGGGATGACCCTTGGGTTTGTCTTTGGGTGTTACCTTGAGTGTCTCTCTGTGTCGCTATCTGTTACAGTCTCCTAAAGTATCCTCCTAAAGTCACCTCCTAACGTCCATCCTAAAGCCAACACCTAAAGCCTACACCTAAAGACCCATCAAGTCAACGCCTATCTTAAAGTTTAAAAATAAAGACCAGACCTAAAGACCAAACCTAAAGACACTACATAAAGACCAGACCTAAAGACGCCTAGTTGTTa                                                           >  1:395276/1‑301 (MQ=255)`  
                                                                  `c‑cAATCACCTAAAGTCAACCTTCGGTTGACCTTGAGGGTTCCCTAAGGGTTGGGGATGACCCTTGGGTTTGTCTTTGGGTGTTACCTTGAGTGTCTCTCTGTGTCCCTATCTGTTACAGTCTCCTAAAGTATCCTCCTAAAGTCACCTCCTAACGTCCATCCTAAAGCCAACACCTAAAGCCTACACCTAAAGACCCATCAAGTCAACGCCTATCTTAAAGTTTAAACATAAAGACCAGGCCTAAAGACCAGACCTAAAGACACTACATAAAGACCAGACCTAAAGACGCCTTGTTGTTAg                                                          >  1:203321/1‑301 (MQ=255)`  
                                                                    `tataagagacaggTCAACCTTCGGTGGACCTTGAGGGTTCCCTAAGGGTTGGGGATGACCCTTGGGTTTGTCTTTGGGTGTTACCTTGAGTGTCTCTCTGTGTCCCTATCTGTTACAGTCTCCTGAAGTATCCTCCTAAAGTCACCTCCTAACGTCCATCCTAAAGCCAACACCTAAAGCCTACACCTAAAGACCCATCAAGTCAACGCCTATCTTAAAGTTTAAACATAAAGACCAGACCTAAAGACCAGACCTAAAGACACTACATAAAGACCAGACCTAAAGACGCCTTGTTGTTAGc                                                         <  1:91092/289‑1 (MQ=255)`  
                                                                        `cACCTAAAGTCAACCTTCGGTTGACCTTGAGGGTTCCCTAAGGGTTGGGGATGACCTTTGGGTTTGTCTTTTGGTGTTACCTTGAGTGTCTCTCTGTGTCCCTATCTGTTACAGTCTCTTAAAGTATCCTCCTAAAGTCACCTCCTCAGGTCCATCCTAAAGCCAACACCTAAAGCCTACAGCTAAAGACCCATCAAGTCAACGACTATCTTAAAGTTTAAACATAAAGACCAGACCTAAAGACCAGACCTAACGACACGAAATAAGGACCAGACATAAAGACGccctttctcttcaacaa                                                     >  1:284099/1‑286 (MQ=37)`  
                                                                          `ccTAAAGTCAACCTTCGGTTGACCTTGAGGGTTCCCTAAGGGTTGGGGATGACCCTTGGGTTTGTCTTTGGGTGTTACCTTGAGTGTCTCTCTGTGTCCCTATCTGTTACAGTCTCCTAAAGTATCCTACTAAAGTCACCTCCTAACGTCCATCCTAAAGCCAACACCTAAAGCCTACACCTAAAGACCCATCAAGTCAACGCCTATCTTAAAGTTTAAACATAAAGACCAGACCTAAAGACCAGACCTAAATACACTACATAAAGACCAGACCTAAAGACGCCTTGTTGTTAGCCATaaa                                                   >  1:73129/1‑301 (MQ=255)`  
                                                                           `cTAAAGTCAACCTTCGGTTGACCTTGAGGGTTCCCTAAGGGTTGGGGATGACCCTTGGGTTTGTCTTTGGGTGTTACCTTGAGTGTCTCTCTGTGTCCCTATCTGTTACAGTCTCCTAAAGTATCCTCCTAAAGTCACCTCCTAACGTCCATCCTAAAGCCAATACCTAAAGCCTACACCTAAAGACCCATCAAGTCAACGCCTATCTTAAAGTTTAAACATAAAGACCAGACCTAAAGACCAGACCTAAAGACACTACATAAAGACCAGACCTAAAGACGCCTTGTTGTTAGCCATAAAg                                                  <  1:239410/301‑1 (MQ=255)`  
                                                                           `cTAAAGTCAACCTTCGGTTGACCTTGAGGGTTCCCTAAGGGTTGGGGATGACCCTTGGGTTTGTCTTTGGGTGTTACCTTGAGTGTCTCTCTGTGTCCCTATCTGTTACAGTCTCCTAAAGTATCCTCCTAAAGTCACCTCCTAACGTCCATCCTAAAGCCAACACCTAAAGCCTACACCTAAAGACCCATCAAGTCAACGCCTATCTTAAAGTTTAAACATAAAGACCAGACCTAAAGACCAGACCTAAAGACACTACATAAAGACCAGACCTAAAGACGCCTTGTTGTTAGCCATAAAg                                                  <  1:408516/301‑1 (MQ=255)`  
                                                                              `aaGTCAACCTTCGGTTAACCTTGAGGGTTCCCTAAGGGTTGGGGATGACCCTTGGGTTTGTCTTTGGGTGTTACCTTGAGTGTCTCTCTGTGTCCCTATCTGTTACAGTCTCCTAAAGTATCCTCCTAAAGTCACCTCCTAACGTCCATCCTAAAGCCAACACCTAAAGCCTACACCTAAAGACCCATCAAGTCAACGCCTATCTTAAAGTTTAAACATAAAGACCAGACCTAAAGACCAGACCTAAAGACACTACATAAAGACCAGACCTAAAGACGCCTTGTTGTTAGCCATAAAGTGa                                               >  1:139439/1‑301 (MQ=255)`  
                                                                                   `gtataagagacagccTTGATGGTTCCCTAAGGGTTGGGGATGACCCTTTGGTTTGTCTTTGGGTGTTACCTTGAGTGTCTCTCTGTGTCCCTATCTGTTACAGTCTCCTAAAGTATCCTCCTAAAGTCACCTCCTAACGTCCATCCTAAAGCCAACACCTAAAGCCTACACCTAAAGACCCATCAAGTCAACGCCTATCTTAAAGTTTAAACATAAAGACCAGACCTAAAGACCAGACCTAAAGACACTACATAAAGACCAGACCTAAAGACGCCTTGTTGTTAGCCATAAAGTGATAAcc                                          <  1:180790/288‑1 (MQ=255)`  
                                                                                   `aaCCTTCGGTTTACCTTAAAGGTTCACTAAGGGTTGGGGATGACCCTTGGGTTAGTCTTTGGGTGTTACCTTGAGTGTCTCTCTGTGTCCCTATCTGTTACAGTCTCCTAAAGTATCCTCCTAAAGTAACCTCCTAACGTCCAACCTAAAACCAACACCTAAAGCCTACACCTAAAGACCCATCAAGTCAACGCCGATCTTAAAGTTTAAACATAAAGACCAGACCTAAAGACCAGACCTAAAGACACTACATAAAGACCAGACCTAAAGACGCCTTGTTGTTAGCCATAAAGTGATAAcc                                          <  1:84227/301‑1 (MQ=255)`  
                                                                                          `ggTTGATCTTGAGGGTTCCCTAAGGGTTGGGGATGAACCTTGGGTTTGTATTTGGGTGTTACCTTGAGTGTCTCTCTGTGTCCCTATCTGTTACAGTCTCCTAAAGTATCCTCCTAAAGTCACCTCCTAACGTCCATCCTAAAGCCAACACCTAAAGCCTACACCTAAAGACCCATCAAGTCAACGCCTATCTTAAAGTTTAAACATAAAGACCAGACCTAAAGACCAGACCTAAAGACACTACATAAAGACCAGACCTAAAGACGCCTTGTTGTTAGCCATAAAGTGATAACCTTTAATc                                   <  1:247626/301‑1 (MQ=255)`  
                                                                                          `ggTTGACCTTGAGGGTTCCCTAAGGGTTGGGGATGACCCTTGGTTTTGTCTTTGGGTGTTACCTTGAGTGTCTCTCTGTGTCCCTATCTGTTACAGTCTCCTAAAGTATCCTCCTAAAGTCACCTCCTAACGTCCATCCTAAAGCCAACACCTAAAGCCTACACCTAAAGACCCATCAAGTCAACGCCTATCTTAAAGTTTAAACATAAAGACCAGACCTAAAGACCAGACCTAAAGACACTACATAAAGACCAGACCTAAAGACGCCTTGTTGTTAGCCATAAAGTGATAACCTTTAATc                                   <  1:290296/301‑1 (MQ=255)`  
                                                                                          `ggTTGACCTTGAGGGTTCCCTAAGGGTTGGGGATGACCCTTGGGTTTGTCTTTGGGTGTTACCTTGAGTGTCTCTCTGTGTCCCTATCTGTTACAGTCTCCTAAAGTATCCTCCTAAAGTCACCTCCTAACGTCCATCCTAAAGCCAACACCTAAAGCCTACACCTAAAGACCCATCAAGTCAACGCCTATCTTAAAGTTTAAACATAAAGACCAGACCTAAAGACCAGACCTAAAGACACTACATAAAGACCAGACCTAAAGACGCCTTGTTGTTAGCCATAAAGTGATAACCTTTAATc                                   <  1:381494/301‑1 (MQ=255)`  
                                                                                          `ggTTGACCTTGAGGGTTCCCTAAGGGTTGGGGATGACCCTTGGGTTTGTCTTTGGGTGTTACCTTGAGTGTCTCTCTGTGTCCCTATCTGTTACAGTCTCCTAAAGTATCCTCCTAAAGTCACCTCCTAACGTCCATCCTAAAGCCAACACCTAAAGCCTACACCTAAAGACCCATCAAGTCAACGCCTATCTTAAAGTTTAAACATAAAGACCAGACCTAAAGACCAGACCTAAAGACACTACATAAAGACCAGACCTAAAGACGCCTTGTTGTTAGCCATAAAGTGATAACCATTAATc                                   <  1:121636/301‑1 (MQ=255)`  
                                                                                          `ggTTGACCTTGAGGGGTCCCTAAGGGTTGGGGAGGACCCTTGGGTTTGTCTTTGGGTGGTACCTTGAGCGTCTCTCCGTGTCCCTATCTGTTACAGTCTCCTAAAGTATCCTCCTAAAGGCACCTCCTAACGTCCATCCTAAAGCCAACACCTAAAGCCTAGACCTAAAGACCCATCAAGTCAACGCCTATCTTAAAGTTTAAACATAAAGACCAGACCTAAAGACCAGACCTAAAGACACTACATAAAGACCAGACCTAAAGACGCCTTGTTGTTAGCCATAAAGTGATAACCTTTAATc                                   <  1:304554/301‑1 (MQ=255)`  
                                                                                          `atgtgtataagagacagcccTAAGTGTTGGGGATGACCCTTGGGTTTGTCTTTGGGTGTTACCTTGAGTGTCTCTCTGTGTCCCTATCTGTTACAGTCTCCTAAAGTATCCTCCTAAAGTCACCTCCTAACGTCCATCCTAAAGCCAACACCTAAAGCCTACACCTAAAGACCCATCAAGTCAACGCCTATCTTAAAGTTTAAACATAAAGACCAGACCTAAAGACCAGACCTAAAGACACTACATAAAGACCAGACCTAAAGACGCCTTGTTGTTAGCCATAAAGTGATAACCTTTAATc                                   <  1:137544/284‑1 (MQ=255)`  
                                                                                           `gTTGACCTTGAGGGTTCCCTAAGGGTTGGGGATGACCCTTGGGTTTGTCTTTGGGTGTTACCTTTAGTGTCTCTCTGTGTCCCTATCTGTTACAGTCTCCTAAAGTATCCTCCTAAAGTCACCTCCTAACGTCCATCCTAAAGCCAACACCTAAAGCCTACACCTAAAGACCCATCAAGTCAACGCCTATCTTAAAGTTTAAACATAAAGACCAGACCTAAAGACCAGACCTAAAGACACTACATAAAGACCAGACCTAAAGACGCCTTGTTGTTATCCATAAAGTGATAACCTTTAATCa                                  <  1:309786/301‑1 (MQ=255)`  
                                                                                                `ccTTTAGGTTTCCCTCAGGGTTGGGGATGACCCTTGGATTTGTCTTTAGGTGTTACCTTGAGTGTCTCTCTGTGTCCCTATCTGTTACAGTCTCCTAAAGTATCCTCCTAAAGTCACCTCCTAACGTCCATCCTAAAGCCAACACCTAAAGCCTACACCTAAAGACCCATCAAGTCCACGCCTATCTTAAAGTTTAAACATAAAGACCAGACCTAAAGACCAGACCTAAAGACACTACATAAAGACCAGACCTAAAGACGCCTTGTTGTTAGCCATAAAGTGATAACCTTTAATCATTGTc                             <  1:321534/301‑1 (MQ=255)`  
                                                                                                `ccTTGAGGGTTCCCTAAGGGTTGGGGATGACCCTTGGGTTTGTCTTTGGGTGTTACCTTGAGTGTCTCTCTGTGTCCCTATCTGTTACAGTCTCCTAAAGTATCCTCCTAAAGTCACCTCCTAACGTCCATCCTAAAGCCAACACCTAAAGCCTACACCTAAAGACCCATCAAGTCAACGCCTATCTTAAAGTTTAAACATAAAGACCAGACCTAAAGACCAGACCTAAAGACACTACATAAAGACCAGACCTAAAGACGCCTTGTTGTTATCCATAAAGTGATAAccctgtctcttatac                             >  1:375819/1‑288 (MQ=255)`  
                                                                                                `ccTTGAGGGTTCCCTAAGGGTTGGGGATGACCCTTGGGTTTGTCTTTGGGTGTTACCTTGAGTGTCTCTCTGTGTCCCTATCTGTTACAGTCTCCTAAAGTATCCTCCTAAAGTCACCTCCTAACGTCCATCCTAAAGCCAACACCTAAAGCCTACACCTAAAGACCCATCAAGTCAACGCCTATCTTAAAGTTTAAACATAAAGACCAGACCTAAAGACCAGACCTAAAGACACTACATAAAGACCAGACCTAAAGACGCCTTGTTGTTAGCCATAAAGTGATAACCTTTAATCAttttt                             >  1:195901/1‑298 (MQ=255)`  
                                                                                                `cagtGAGGGTTCCCTAAGGGTTGGGGATGACCATTGGGTTTGTCTTTGGGTTGTACCTTGAGTGTCTTTCTGTGTCCCTATCTGTTCCATTCTCCTAAAGTATCCTCCTAAAGTCACCTCCTAACGTACATCCTAAAGCCAACACCTAAAGCCTACACCTAAAGACCCATCAAGTCAACGCCTATCTTAAAGTTTAAACATAAAGACCAGACCTAAAGACCAGACCTAAAGACACTACATAAAGACCAGACCTAAAGACGCCTTGTTGTTAGCCATAAAGTGATAACCTTTAATCATTGTc                             <  1:485777/298‑1 (MQ=255)`  
                                                                                                 `cgtGAGGGTTCCCTAAGGGTTGGGGATGACCCGTGGGTTTGTCTTTGGGTGTTACCTTGAGTGTCTCTCTGTGTACCTATCTGTTACAGTCTCCTAAAGTATCCTCCTAAAGTCACCTCCTAACGTCCATCCTAAAGCCAACACCTAAAGCCTACACCTAAAGACCCATCAAGTCAACGCCTATCTTAAAGTTTAAACATAAAGACCAGACCTAAAGACCAGACCTAAAGACACTACATAAAGACCAGACCTAAAGACGCCTTGTTGTTAGCCATAAAGTGATAACCTTTAATCATTGTCt                            <  1:167826/299‑1 (MQ=255)`  
                                                                                                     `aGGGTACCTAAAGGGTGGGGAGTAACCTTAGGGTTGGTTTTTGGGT‑TTACCTTTGAAATCTCACTGGATTCCTTACAATTTCAGTCTACCAAAGTATACTCCTAAAGACACCTCCTAACGTCCATCCTAAAGCCAACACCTAAAGCCTACACCTAAAGACCCATCAAGTCAACGCCTATCTTAAAGTTTAAACATAAAGACCAGCCCTAAAGACCAGACCTAAAAACACTACATAAAGACCAGACCTAAAGACGCCTTGTTGTTAGCCATAAAGTGATAACCTTTAATCATTGTCTttatt                       <  1:334377/301‑1 (MQ=255)`  
                                                                                                       `ggTTCCCTCAGGGTTGGGGATGACCCTTGGGTTTGTCTTTGGGTGTTACCTTGAGTGTCTCTCTGTGTCCCTATCTGTTACAGTCTCCTAAAGTATCCTCCTAAAGTCACCTCCTAACGTCCATCATAAAGCCAACACCTAAAGCCTACACCTAAAGACCCATCAAGTCAACGCCTATCTTAAAGTTTAAACATAAAGACCAGACCTAAAGACCAGACCTAAAGACACTACATAAAGACCAGACCTAAAGACGCCTTGTTGTTAGCCATAAAGTGATAACCTTTAATCATTGTCTttattt                      >  1:392271/1‑300 (MQ=255)`  
                                                                                                       `ggTTCCCTAAGGGTAGGGGATGAACCTGGGGTTTGTCTTTGGGTGTTAACTTGAGTGACTCTCTGTGTCCCTATCTGTTACAGTCTACTAAAGGAACATCCTAAAGTAACCTCCTAACGTGCATCCTAAAGCCAACACCTAAAGCCTACACCTAAAGACCCATCAAGTCAACGCCTAGCTTAAAGTTTAAACATAAAGACAAGACCTAAAGACCAGACCTAAAGACACTACATAAAGACCAGACCTAAAGACGCCTTGTTGTTAGCCATAAAGTGATAACCTTTAATCATTGTCTttatta                      <  1:315847/301‑1 (MQ=255)`  
                                                                                                          `tCCCTAAGGGTTGGGGATGACCCTTGGGTTTGTCTTTGGGTGTTACCTTGAGTGTCTCTCTGTGTCCATATCTGTTACAGTCTCCTAAAGTATCCTCCTAAAGTCACCTCCTAACGTCCATCCTAAAGCCAACACCTCAAGCCTACACCTAAAGACCCATCAAGTCAACGCCTATCTTAAAGTTTAAACATAAAGACCAGACCTAAAGACCAGCACTAAAGACTATACATAACGACCAGACCTAAAGACGCCTTGTTGTTAGCCATAAAGTGATAAccctgtctcttatacacatctccga                   >  1:14884/1‑278 (MQ=39)`  
                                                                                                           `cccTAAGGGTTGGGGATGACCCTTGGGTTTGTCTTTGGGTGTTACCTTGAGTGTCTCTCTGTGTCCCTATCTGTTACAGTCTCCTAAAGTATCCTCCTAAAGTCACCTCCTAACGTCCATCCTAAAGCCAACACCTAAAGCCTACACCTAAAGACCCATCAAGTCAACGCCTATCTTAAAGTTTAAACATAAAGACCAGACCTAAAGACCAGACCTAAAGACACTACATAAAGACCAGACCTAAAGACGCCTTGTTGTTAGCCATAAAGTGATAACCTTTAATCATTGTCTTTATTAATAc                  <  1:273320/301‑1 (MQ=255)`  
                                                                                                           `cccTAAGGGTTGGGGATGACCCTTGGGTTTGTCTTTGGGTGTTACCTTGAGTGTCTCTCTGTGTCCCTATCTGTTACAGTCTCCTAAAGTATCCTCCTAAAGTCACCTCCTAACGTCCATCCTAAAGCCAACACCTAAAGCCTACACCTAAAGACCCATCAAGTCAACGCCTATCTTAAAGTTTAAACATAAAGACCAGACCTAAAGACCAGACCTAAAGACACTAAATAAAGACCAGACCTAAAGAAGCCTTGTTGTTAGCCATAACGGGATAACCTTTAATCATTTACGTTATTaaaaa                  >  1:400049/1‑298 (MQ=255)`  
                                                                                                           `cccTAAGGGTTGGGGATGACCCTTGGGTTAGTCTTTGGGTGTTACCTTGAGTCTCTCTCTGTGCTCCTCTCCGTTACAGCCTTCTAAAGTATCCTTCTAAAGTTACCTCCTAACGTCCATCCTAAAGTCAACAACTAAAGCCTACACCTAAAGACCCATCAAGTCAACGTATATATTAAAGTTTAAACATAATTACCAGAACTAAAGACCAGACCTAAAGACACTACAATAACACCAGACCTAAAGACGCCTTGTTGTTAGCCATAAAGTGATAAccacaaatccagtatctaagacaact                  >  1:126728/1‑277 (MQ=255)`  
                                                                                                              `aaaGGGTTGGGGATGACCCTTGGGTTTGTCTTTGGGTGTTACCTTGAGTGTCTCTCTGTGTCCCTATCTGTTACAGTCTCCTAAAGTATCCTCATAAAGTCACCTCCTAACGTCCATCCTAAAGCCAACACCTAAAGCCTACACCTAAAGACCCATCAAGTCAACGCCTATCTTAAAGTTTAAACATAAAGACCAGACCTAAAGACCAGACCTAAAGACACTACATAAAGACCAGACCTAAAGACGCCTTGTTGTTAGCCATAAAGTGATAACCTTTAATCATTGTCTTTATTAATACAAc               <  1:454632/300‑1 (MQ=255)`  
                                                                                                                `tgggTTGGGGATGACCCTTTGGTTTGGTTGTGGGTGGTACCTTGAGTTGCTCTCTGTGTCCCTATCTGTTACAGTCTCCTAAAGTATCCCCCTAAAGTCACCTCCTAACGTCCATCCTAAAGCCAACACCTAAAGCCTACACCTAAAGACCCATCAAGTCAACGCCTATCTTAAAGTTTAAACATAAAGACCAGACCTAAAGACCAGACCTAAAGACACTACATAAAGACCAGACCTAAAGACGCCTTGTTGTTAGCCATAAAGTGATAACCTTTAATCATTGTCTTTATTAATACAACTc             <  1:280434/300‑1 (MQ=255)`  
                                                                                                                `ggggTTGGGGATGACCCTTGGGTTTGTCTTTGGGTGTTACCTTGAGTGTCTCTCTGTGGCCCTATCTGTTACAGTCTCCTAAAGTATCCTCCTAAAGTCACCTCCTAACGTCCATACTAAAGCCAACACCTAAAGCCTAAACCTAAAGACCCATCAAGTCAACGCCTATCTTAAAGTTTAAACATAAAGACCAGACCTAAAGACCAAACCTAAAGACACTACATAAAGACCAGACCTAAAGACGCCTTGTTGTTAGCCATAAAGTGATAACCTTTAATCATTGTCTTTATTAATACAACTc             <  1:503312/300‑1 (MQ=255)`  
                                                                                                                 `gggTTGTGGATGACCCTTGGGTTTGTCTTTTGGTGTTACCTTGAGTGTCTCTCTGTGTCCCTATCTGTTACAGTCTCCTAAAGTATCCTCCTAAAGTCACCTCCTAACGTCCATCCTAAAGCCAACACCTAAAGCCTACACCTAAAGACCCATCAAGTCAACGCCTATCTTAAAGTTTAAACATAAAGACCAGAACTAAAGACCAGACCTAAAGACACTACATAAAGACCAGACCTAAAGACGCCTTGTTGTTCGCCATAAAGTGATAACCTTTAATCATTGTTTTTATTAATACAAATcc            >  1:267047/1‑300 (MQ=255)`  
                                                                                                                 `gggTTGGGGATGACCCTTGGGTTTGTCTTTGGGTGTTACCTTGAGTGTCTCTCTGTGTCCCTATCTGTTACAGTCTCCTAAAGTATCCTCCTAAAGTCACCTCCTAACGTCCATCCTAAAGCCAACACCTAAAGCCTACACCTAAAGACCCATCAAGTCAACGCCTATCTTAAAGTTTAAACATAAAGACCAGACCTAAAGACCAGACCTAAAGACACTACATAAAGACCAGACCTAAATACGCCTTGTTGTTAGCCATAAAGTGATAACCTTTAATAATTGTCTTTATTAATAcctgtct            >  1:270029/1‑295 (MQ=255)`  
                                                                                                                   `gTTGGGGATGACCCTTGGGTTTGTCTTTGGGTGTTACCTTGAGTGTCTCTCTGTGTCCCTATCTGTTACAGTCTCCTAAAGTATCCTCCTAAAGTCACCTCCTAACGTCCATCCTAAAGCCAACACCTAAAGCCTACACCTAAAGACCCATCAAGTCAACGCCTATCTTAAAGTTTAAACATAAAGACCAGACCTAAAGACCAGACCTAAAGAAACTACATAAAGACCAGACCTACAGACGCCTTGTTGTTAGCCATAAAGTGATAAACTTTAATCATTGTCTTTATTAATACAACTCACt          >  1:346105/1‑301 (MQ=255)`  
                                                                                                                     `tgtggATGACCCTTGGGTTTGTCTTTGGGTGTTACCTTGAGTGTCTCTCTGTGTCCCTATCTGTTACAGTCTCCTAAAGTATCCTCCTAAAGTCACCTCCTAACGTCCATCCTAAAGCCAACACCTAAAGCCTACACCTAAAGACCCATCAAGTCAACGCCTATCTTAAAGTTTAAACATAAAGACCAGACCTAAAGACCAGAACTAAAGACACTACATAAAGACCAGACCTAAAGACGCCTTGTTGTTAGCCATAAAGTGATAACCTTTAATCATTGTCTTAATTAATACAACTCACtat        <  1:161114/298‑1 (MQ=255)`  
                                                                                                                       `gggATGACCCTTGGGTTTGTCTTTGGGTGTTACCTTGAGTGTCTCTCTGTGTCCCTATCTGTTACAGTCTCCTAAAGTATCCTCCTAAAGTCACCTCCTAACGTCCATCCTAAAGCCAACACCTAAAGCCTACACCTAAAGACCCATCAAGTCAACGCCTATCTTAAAGTTTAAACATAAAGACCAGACCTAAAGACCAGACCTAAAGACACTACATAAAGACCAGACCTAAAGACGCCTTGTTGTTAGCCATAAAGTGATAACCTTTAATCATTGTCTTTATTAATACAACTCACTATaa      >  1:199690/1‑301 (MQ=255)`  
                                                                                                                        `ggATGACCCTTGGGTTTGTCTTTGGGTGTTACCTTGAGTGTCTCTCTGTGTCCCTATCTGTTACAGTCTCCTAAAGTATCCTCCTAAAGTCACCTCCTAACGTCCATCCTAAAGCCAACACCTAAAGCCTACACCTAAAGACCCATCAAGTCAACGCCTATCTTAAAGTTTAAACATAAAGACCAGACCTAAAGACCAGACCTAAAGACACTACATAAAGACCAGACCTAAAGACGCCTTGTTGTTAGCCATAAAGTGATAACCTTTAATCATTGTCTTTATTAATACAACTCACTATAAg     <  1:156700/301‑1 (MQ=255)`  
                                                                                                                         `gATGACCCTTGGGTTTGTCTTTGGGTGTTACCTTGAGTGTCTCTCTGTGTCCCTATCTGTTACAGTCTCCTAAAGTATCCTCCTAAAGTCACCTCCTAACGTCCATCCTAAAGCCAACACCTAAAGCCTACACCTAAAGACCCATCAAGTCAACGCCTATCTTAAAGTTTAAACATAAAGACCAGACCTAAAGACCAGACCTAAAGACACTACATAAAGACCAGACCTAAAGACGCCTTGTTGTTAGCCATAAAGTGATAACCTTTAATCATTGTCTTTATTAATACAACTCACTATAAgg    <  1:388855/301‑1 (MQ=255)`  
                                                                                                                         `gATGACCCTTGGGTTTGTCTTTGGGTGTTACCTTGAGTGTCTCTCTGTGTCCCTATCTGGTACAGTCTCCTAAAGTATCCTCCTAAAGTCACCTCCTAACGTCCATCCTAAAGCCAACACCTAAAGCCTACACCTAAAGACCCATCAAGTCAACGCCTATCTTAAAGTTTAAACATAAAGACCAGACCTAAAGACCAGACCTAAAGACACTACATAAAGACCAGACCTAAAGACGCCTTGTTGTTAGCCATAAAGTGATAACCTTTAATCATTGTCTTTATTAATACAACTCACTATAAgg    <  1:277262/301‑1 (MQ=255)`  
                                                                                                                         `gATGACCCTTGGGTTTGTCTTTGGGTGTTACCCTGAGTGGCTATCTGTGTCCCTATCTGTTACAGTCTCCTAAAGTATCCTCCTAAAGTCACCTCCTAACGTCCATCCTAAAGCCAACAACGAAAGCCTACACCTAAAGACCCATCAAGTCAACGCCTATCTTAAAGTTTAAACATAAAGACCAGACCTAAAGACCAGACCTAAAGACACTACATAAAGACCAGACCTAAAGACGCCTTGTTGTTAGCCATAAAGTGATAACCTTTAATCATTGTCTTTATTAATACAACTCACTATAAgg    <  1:45838/301‑1 (MQ=255)`  
                                                                                                                          `aTGACCCTTGGGTTTGTCTTTGGGTGTTACCTGGAGTGTCTCACTGTGTCACTAGCTGGTACAGTCTCCTAAAGTATCCAACTAAAGTCAACTCCTAACGTCCATCCTATAGCCAACACCTAAAGCCTACACCGAAAGACCCATCAAATCAACGCCTATCTTAAAGTTTAAACATAAAGACCAGACCTAAATCCCAGACATAAAGACACTACATAAAGACCAGACCTAAAGTCGCCTTGTTGTTAGCCATAAAGTGATAACCTTTAATCATTGTCTTTATTAATACAACTCACTATAAGga   <  1:43120/301‑1 (MQ=255)`  
                                                                                                                           `tGACCCTTGGGTTTGTCTTTGGGTGTTACCTTGAGTGTCTCTCTGTGTCCCTATCTGTTACAGTCTCCTAAAGTATCCTCCTAAAGTCACCTCCTAACGTCCATCCTAAAGCCAACACCTAAAGCCTACACCTAAAGACCCATCAAGTCAACGCCTATCTTAAAGTTTAAACATAAAGACCAGACCTAAAGACCAGACCTAAAGACACTACATAAAGACCAGACCTAAAGACGCCTTGTTGTTAGCCATAAAGTGATAACCTTTAATCATTGTCTTTATTAATACAACTCACTATAAGgag  <  1:492367/301‑1 (MQ=255)`  

                                                                                                                           `|`                                                                                                                                                                                                                                                                                                              
`‑‑‑‑‑‑‑‑‑‑‑‑‑‑TCTCACAGTGTACGGACCTAAAGTTCCCCCATAGGGGGTACCTAAAGCCCAGC‑CAATCACCTAAAGTCAACCTTCGGTTGACCTTGAGGGTTCCCTAAGGGTTGGGGATGACCCTTGGGTTTGTCTTTGGGTGTTACCTTGAGTGTCTCTCTGTGTCCCTATCTGTTACAGTCTCCTAAAGTATCCTCCTAAAGTCACCTCCTAACGTCCATCCTAAAGCCAACACCTAAAGCCTACACCTAAAGACCCATCAAGTCAACGCCTATCTTAAAGTTTAAACATAAAGACCAGACCTAAAGACCAGACCTAAAGACACTACATAAAGACCAGACCTAAAGACGCCTTGTTGTTAGCCATAAAGTGATAACCTTTAATCATTGTCTTTATTAATACAACTCACTATAAGGAG  >  NC_001604.1/1‑409`

  

Alignment Legend

Aligned base mismatch/match (shaded by quality score): `ATCG/ATCG` `< 3 ≤` `ATCG/ATCG` `< 10 ≤` `ATCG/ATCG` `< 20 ≤` `ATCG/ATCG` `< 33 ≤` `ATCG/ATCG` `< 38 ≤` `ATCG/ATCG`

Unaligned base: `atcg`    Masked matching base: `atcg`    Alignment gap: `‑`    Deleted base: `‑`
